# Epidemiology Characteristics of *Streptococcus pneumoniae* From Children With Pneumonia in Shanghai: A Retrospective Study

**DOI:** 10.3389/fcimb.2019.00258

**Published:** 2019-07-18

**Authors:** Wantong Zhao, Fen Pan, Bingjie Wang, Chun Wang, Yan Sun, Tiandong Zhang, Yingying Shi, Hong Zhang

**Affiliations:** Department of Clinical Laboratory, Shanghai Children's Hospital, Shanghai Jiaotong University, Shanghai, China

**Keywords:** *Streptococcus pneumonia*, serotypes, antibiotic resistance, pneumococcal pneumonia, pneumococcal conjugate vaccine, virulence, children

## Abstract

**Background:** *Streptococcus pneumoniae* is the most common pathogen causing death in children under 5 years old. This retrospective surveillance aimed to analyze serotype distribution, drug resistance, virulence factors, and molecular characteristics of pneumonia isolates from children in Shanghai, China.

**Methods:** A total of 287 clinical pneumococcal isolates were collected from January to December in 2018 and were divided into community-acquired pneumonia (CAP) and healthcare-associated pneumonia (HAP) two groups according to where someone contracts the infection. All isolates were serotyped by multiplex sequential PCR and antimicrobial susceptibility testing was performed using E-test or disk diffusion method. The molecular epidemiology was analyzed using multilocus sequence typing and seven housekeeping genes were sequenced to identified the sequence types (STs). In addition, we investigated the presence of virulence genes via PCR.

**Results:** The most common serotypes were 19F, 6A, 19A, 23F, 14, and 6B, and the coverage rates of the 7-, 10- and 13-valent pneumococcal conjugate vaccines were 58.9, 58.9, and 80.5%, respectively. More PCV13/non-PCV7 serotypes and higher rate of penicillin non-susceptible *S. pneumoniae* were seen in HAP. Molecular epidemiological typing showed a high level of diversity and five international antibiotic-resistant clones were found, including Taiwan^19F^-14, Spain^23F^-1, Spain^6B^-2, Taiwan^23F^-15 and Sweden^15A^-25. No significant difference was observed in the presence of virulence genes among the isolates obtained from CAP and HAP. All of the *S. pneumoniae* isolates carried *lytA, ply, psaA, pavA, spxB, htrA*, and *clpP*, and the carriage rate of *nanA* and *piaA* were 96.2 and 99.0%. Conversely, *cps2A, cbpA*, and *pspA* were present in 33.8–44.3% of the isolates.

**Conclusions:** Serotype changes and emerging multidrug-resistant international clones were found in current study. *lytA, ply, psaA, pavA, spxB, htrA*, and *clpP* may be good protein vaccine candidates. Long-term high-quality surveillance should be conducted to assess impact and effectiveness brought by vaccines, and provide a foundation for prevention strategies and vaccine policies.

## Introduction

*Streptococcus pneumoniae* is a frequent colonizer of the human nasopharynx with a colonization rate of 27–65% in children (Weiser et al., 2018), whilst the cause of both invasive pneumococcal disease (including bacteremia, meningitis, etc.) and non-invasive pneumococcal disease such as pneumonia and otitis media under the condition of the immunocompromised or microflora imbalance (GBD 2016 Lower Respiratory Infections, 2018; Weiser et al., 2018). It presents as a burden associated with high morbidity and mortality globally. As the global estimates reported, of all pneumococcal deaths in HIV-uninfected children in 2015, 81% of them died of pneumonia (Wahl et al., 2018). Centers for Disease Control and Prevention of America recommends that pneumonia can be divided into two types according to place where someone contracts the infection, community-acquired pneumonia (CAP) which is defined as when someone develops pneumonia in the community (not in a hospital) and healthcare-associated pneumonia (HAP) which is defined as when someone develops pneumonia during or following a stay in a healthcare facility^1^.

In the lower respiratory infections in 195 countries in 2016, *S. pneumoniae* was estimated to be responsible for 341029 deaths of children younger than 5 years (GBD 2016 Lower Respiratory Infections, 2018). By far, lower respiratory infection incidence and mortality in children is mostly attributed to pneumococcal pneumonia. Vaccines and antibiotics are considered as effective methods against *S. pneumoniae*. Immunizing with vaccines was suggested by WHO to prevent *S. pneumoniae* infections (Pneumococcal vaccines WHO position paper, 2012). A reduction in CAP of > 40% after introduction of PCV7 has also been reported (Falup-Pecurariu, 2012). In Shanghai, pneumococcal vaccines belong to the second category of vaccines and vaccination is given only on an voluntary basis at their own expense, which maybe the cause of low vaccination rate of PCV. On the other hand, antimicrobial therapy is the common anti-infection treatment. However, with the changes in *S. pneumoniae* serotype and antibiotic resistance over time, the current treatment options are constantly being adjusted as well. The epidemiological data of pneumococcus on children with pneumonia in Shanghai is scarce at present. In this study, we aimed to analyze serotype distribution, antibiotic resistance, virulence factors and molecular characteristics of pneumonia isolates identified from children in Shanghai to provide data support for development of pneumococcal infection prevention strategies and vaccines.

## Materials and Methods

### Clinical Isolates and Population

The retrospective surveillance was conducted at Shanghai Children's Hospital, which is the first specialist children's hospital in China, with about 2.5 million outpatients visiting and 44,000 hospitalized each year. A total of 287 *S. pneumoniae* isolates were collected from patients diagnosed with pneumonia between January and December in 2018. CAP included the isolates obtained from an outpatient or collected earlier than 48 h after hospitalization, while specimens obtained more than 48 h after admission were included as HAP in this investigation (Sader et al., 2018).

Clinical and epidemiological information was systematically extracted from the medical records, including demographics of the patient, symptoms and findings at hospitalization, underlying, and other potential characteristics. The protocol for present study was approved by the Shanghai Children's Hospital Ethics Committee (Shanghai Jiao Tong University School of Medicine). The retrospective study was to obtain the genus and species of the bacteria and did not affect the patients, the Review Board consequently exempted the informed consent requirements. Only one isolate was collected from each patient. Duplicate strains and patients colonized by bacteria with no clinical symptoms were excluded from the study.

### Microbiology Methods

The pneumococcal isolates analyzed in current study were collected and cultured in line with the need of clinical procedures. Specimens were collected by professional staff or doctors and transported to the department of clinical microbiology within 2 h, which were inoculated onto 5% sheep blood agar plates and incubated at 35°C, 5% CO_2_ for 18–24 h. All isolates were identified by typical colony morphology, optochin assays and confirmed by the matrix-assisted laser desorption ionization-time of flight-mass spectrometry (MALDI-TOF MS; Bruker Daltonik GmbH, Bremen, Germany). Strains identified as *S. pneumoniae* were stored in 40% sterile glycerol broth at −80°C for subsequent analysis.

### Serotyping

*S. pneumoniae* isolates were serotyped by multiplex sequential PCR (MP-PCR), and a primer pair targeting *cpsA* was used as a positive control in each reaction (Pai et al., 2006). Serogroup 6A/B were identified using the method described previously (Jin et al., 2009). If the serotype was not detected by the method mentioned above, the strain was classified as non-typeable. Afterwards, the coverage rates of PCV7, PCV10, and PCV13 were estimated by calculating the percentage of isolates expressed the serotypes included in the vaccines.

### *In vitro* Antimicrobial Susceptibility Testing

Antimicrobial resistance testing of all 287 isolates were determined by E-test and Kirby-Bauer disk tests. In our study, we used E-test assay (AB Biodisk, Solna, Sweden) to measure the minimum inhibitory concentrations (MICs) to penicillin. The susceptibility to clindamycin, erythromycin, linezolid, moxifloxacin, sulfamethoxazole-trimethoprim and vancomycin was assessed using the disk diffusion method (Oxoid Ltd, Basingstoke, UK). All susceptibility tests and results interpretations were performed following the guidelines and criteria established by the Clinical and Laboratory Standard Institute (CLSI) 2018. The quality-control strain was *S. pneumoniae* ATCC 49619, which included in each set of tests to ensure the reliability of the results. Isolates resistant to three or more kinds of antibiotics tested were defined as MDR *S. pneumoniae* in this study.

### Multilocus Sequence Typing

To determine the STs of the isolates, multilocus sequence typing (MLST) analysis was carried out in accordance with the *S. pneumoniae* MLST protocol (Enright and Spratt, 1998). In our experiment, we used the seven housekeeping genes (*aroE, gdh, gki, recP, spi, xpt, and ddl*), which were amplified by PCR using primers previously described (Enright and Spratt, 1998). The internal fragments amplified were sequenced on both strands by the Sanger method using the primers that were used for the initial amplification. Alleles and sequence types (STs) were confirmed by querying the pneumococcal MLST database (http://pubmlst.org/spneumoniae/). The STs obtained were then compared with Pneumococcal Molecular Epidemiology Network (PMEN) clones (http://www.pneumogen.net/pmen/). STs that were different from any known ST were submitted for new name assignment. The relatedness between the isolates was constructed by eBURST version3.0 software. Strains were assigned to a clonal complex (CC) based on the stringent group definition of six of seven shared alleles (Feil et al., 2004).

### Detection of Virulence Genes

A total of 12 genes related to virulence were detected by PCR using published primers (Ibrahim et al., 2004; Shakrin et al., 2014; Bryant et al., 2016; Kang et al., 2016), including capsular polysaccharide (*cps2A*), autolysin (*lytA*), pneumococcal surface protein A (*pspA*), choline binding protein A (*cbpA*), neuraminidase (*nanA*), ion transporters (*piaA*), pneumolysin (*ply*), pneumococcal surface adhesin A (*psaA*), pneumococcal adherence and virulence factor A (*pavA)*, pyruvate oxidase (*spxB*), serine protease high-temperature requirement A (*htrA*), and caseinolytic protease (*clpP*). The PCR products were analyzed by gel electrophoresis and sequencing. The positive products were confirmed by comparing to the online database via BLAST.

### Statistical Analysis

Antibiotic resistance was analyzed with the WHONET 5.6 software, while SPSS 24.0 was used for statistical analysis. Chi-square test or Fisher's exact test were used for significance comparison of categorical data, whereas *t*-test or Rank-sum test were used for comparing quantitative data. P <0.05 was considered to be statistically significant.

## Results

### Demographic and Clinical Characteristics

The total collection presented 287 *S. pneumoniae* isolates causing pneumonia, of which 243 from CAP and 44 from HAP. As was shown in Table 1, 90.9% of these strains (261/287) were isolated from children aged 0–5 years old and the male to female sex ratio was 1.3:1. Number of cases diagnosed in summer (during June to August) were a little lower than other seasons. The common chronic diseases in this study are congenital heart disease (28/287) and asthma (14/287). Concurrent infection was noted in 48.8% patients. No children were vaccinated with pneumococcal conjugate vaccine (PCV) in current study. In the aggregate, discharge data showed that all patients had a favorable prognosis. CAP was the most common in the respiratory department whereas the rate of HAP in the gastroenterology dept is higher. There is no statistical difference in the sex, age, season, prognosis and other clinical and demographic characteristics between CAP and HAP.

**Table 1:** Clinical and demographic characteristics of children with CAP (*n* = 243) and HAP (*n* = 44) in Shanghai.

	**CAP (*n* = 243)**	**HAP (*n* = 44)**	**Total (*n* = 287)**	***%***	***P***
**Sex**					0.202
Male	135 (55.6)	29 (65.9)	164	57.1	
Female	108 (44.4)	15 (34.1)	123	42.9	
**Age (months)**					0.881
<12	49 (20.2)	11 (25.0)	60	20.9	
12–24	66 (27.2)	12 (27.3)	78	27.2	
24–60	105(43.2)	18 (40.9)	123	42.9	
>60	23 (9.5)	3 (6.8)	26	9.1	
**Disease-onset season**					0.992
December-February	59 (24.3)	11 (25.0)	70	24.4	
March-May	69 (28.4)	13 (29.5)	82	28.6	
June-August	49 (20.1)	8 (18.2)	57	19.9	
September-November	66 (27.2)	12 (27.3)	78	27.2	
**Chronic disease**					
Congenital heart disease	20 (8.2)	8 (18.2)	28	9.8	0.077
Asthma	14 (5.8)	0	14	4.9	0.211
**Concurrent infection**					
*Mycoplasma pneumoniae*	61 (25.1)	9 (20.5)	70	24.4	0.509
*Rhinovirus*	23 (9.5)	2 (4.5)	25	8.7	0.439
*Respiratory syncytial virus*	14 (5.8)	2 (4.5)	16	5.6	1.000
*Influenza virus*	15 (6.2)	0	15	5.2	0.185
*Epstein-Barr virus*	8 (3.3)	1 (2.3)	9	3.1	1.000
*Cytomegalovirus*	5 (2.1)	0	5	1.7	1.000
**Wards of hospitalization**					
Respiratory medicine	181 (74.5)	25(56.8)	206	71.7	0.017
Priority ward	42 (17.3)	9 (20.5)	51	17.8	0.613
Gastroenterology	5 (2.1)	5 (11.4)	10	3.5	0.008
ICU	5 (2.1)	2 (4.5)	7	2.4	0.650
Others^*^	10 (4.1)	3 (6.8)	13	4.5	0.690
**Fever (days), median (IQR)**	2 (1-4)	2 (1-4)	-	-	0.657
**Antibiotic (days), median (IQR)**	7.21 (6-8)	6.69 (5-7.5)	-	-	0.395
**Hospitalization (days), median (IQR)**	7 (6-8)	7 (6-9)	-	-	0.112
**Cure**	243 (100)	44 (100)	287	100	

* *Other wards of hospitalization, including Neurology, Cardiology and Neonatology, Otolaryngology-Head and Neck Surgery*.

### Serotype Distribution and Vaccine Coverage

**Figure 1:**
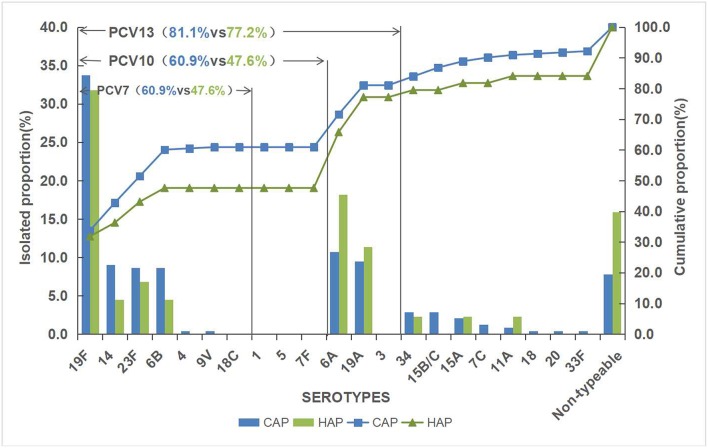
Serotype distribution of pneumococcal strains isolated in Shanghai in 2018.

Of the 287 pneumococcal isolates, 261 isolates (90.9%) were successfully serotyped and 19F (33.4%) was the most common serotype, followed by 6A (11.8%), 19A (9.8%), 23F (8.4%), 14 (8.4%), 6B (8.0%), 34 (2.8%), 15B/C (2.4%), and 15A (2.1%). Other uncommon serotypes were detected in fewer than five strains each, which included serotype 7C (3), 11A (3), 20 (1), 4 (1), 33F (1), 9V (1), and 18 (1). The rest 26 isolates were classified as non-typeable. The serotype distribution of pneumococcal strains isolated is shown in Figure 1.

The overall vaccine coverage rates of PCV7, PCV10, and PCV13 serotypes were 58.9, 58.9, and 80.5%, respectively. HAP had a lower vaccine serotype coverage rate than CAP. A higher rate of PCV13/non-PCV7 serotypes was noticed in HAP. Simultaneously, serotype 4, 9V, 15B/C, 7C, 18, 20 and 33F were only observed in CAP.

### Antibiotic Susceptibility

The total prevalence of penicillin non-susceptible *S. pneumoniae* (PNSP) was 31.7% including penicillin-intermediate *S. pneumoniae* (PISP, 26.8%) and penicillin-resistant *S. pneumoniae* (PRSP, 4.9%) (Table 2). Most strains showed high resistance to erythromycin and clindamycin (>95%). No drug-resistant strains to linezolid, moxifloxacin and vancomycin were observed in this study. In addition, resistance to sulfamethoxazole-trimethoprim was seen in 76.7% of isolates.

**Table 2:** Antimicrobial resistance of pneumococcal isolates from children with CAP (*n* = 243) and HAP (*n* = 44).

**Agent (%)**	**CAP (*N* = 243)**			**HAP (*N* = 44)**			**TOTAL (*N* = 287)**		
	**PSSP (*n* = 168)**	**PISP (*n* = 65)**	**PRSP (*n* = 10)**	**PSSP (*n* = 28)**	**PISP (*n* = 12)**	**PRSP (*n* = 4)**	**PSSP (*n* = 196)**	**PISP (*n* = 77)**	**PRSP (*n* = 14)**
SXT	69.0	92.3	90.0	67.9	100	100	68.9	93.5	92.9
ERY	99.4	96.9	100	100	100	100	99.5	97.4	100
CLI	98.8	96.9	80.0	100	100	75.0	99	97.4	78.6
VAN	0	0	0	0	0	0	0	0	0
LZD	0	0	0	0	0	0	0	0	0
MXF	0	0	0	0	0	0	0	0	0

*SXT, sulfamethoxazole-trimethoprim; ERY, erythromycin; CLI, clindamycin; VAN, vancomycin; LZD, linezolid; MXF, moxifloxacin*.

Approximately 74.9% (215/287) of the isolates were defined as MDR. Resistance of pneumococcus to the agents above among different serotypes was also assessed and it was found that the antibiotic resistance varied by serotype. Serotypes 19F, 19A, and 23F prevailed in PNSP isolates, and almost all 19F isolates were resistant to sulfamethoxazole-trimethoprim. PCV13 covered 85.1% (183/215) of the MDR strains, which was higher than that for PCV7 (61.4%, 132/215). Emerging serotypes (11A, 15B/C, 18, 20, 34, 7C, and 9A) accounted for 8.4% MDR. Compared with CAP, there were higher rates of PNSP (30.9 vs. 36.4%) and MDR (74.5% vs. 77.3%) in HAP.

### MLST

**Figure 2:**
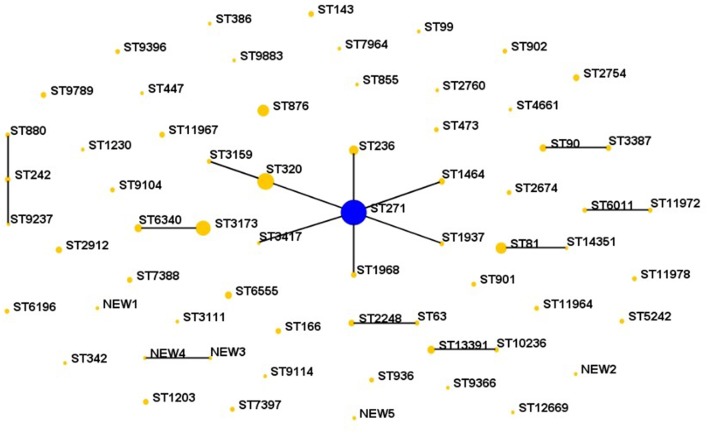
Population snapshot of 287 *S. pneumoniae* revealed by eBURST analysis.

Sixty-four STs were identified by MLST analysis among the 287 isolates. The five predominant STs were ST271 (*n* = 72, 25.1%), ST320 (*n* = 30, 10.5%), ST3173 (*n* = 24, 8.4%), ST876 (*n* = 14, 4.9%), and ST81 (*n* = 13, 4.5%), which were mainly related to serotype 19F, 19A, 6A/B, 14, and 23F, respectively. Nine clonal complexes and 39 singletons were obtained using eBURST version3.0 software analysis for the homology relationship between these STs (Figure 2). Among the 9 CCs, the most prevalent clonal complex CC271 (including ST271, ST236, ST320, ST1968 etc.) accounted for 42.9% (123/287) of the isolates, followed by CC3173 (10.5%, 30/287) and CC81 (4.9%, 14/287).

Comparing the isolates with the PMEN clones (at least 6 of 7 MLST alleles shared), five international antibiotic-resistant clones were found in this study, including Taiwan^19F^-14, Spain^23F^-1, Spain^6B^-2, Taiwan^23F^-15 and Sweden^15A^-25. The isolates belonging to these international clones or their single locus variants (SLVs) made up of 40.8% of all strains. The dominating of the five international clones was Taiwan^19F^-14. Furthermore, CC271 related to the Taiwan^19F^-14 was mainly associated with the serotype 19 group, 88 of which were serotype 19F and 25 were 19A. Sweden^15A^-25 was also identified in this study and this group of isolates included two STs, ST63 (*n* = 2) and SLV ST2248 (*n* = 4), with serotypes 15A and 14, respectively. What's more, one new *aroE* allele and five new STs were found in our study. There were no significant differences in the major STs and distribution between HAP and CAP.

### Presence and Distribution of Virulence Genes

The presence of virulence genes did not show any significant difference among the isolates obtained from CAP and HAP (Table 3). Irrespective of the source of the isolation, all isolates carried *lytA, ply, psaA, pavA, spxB, htrA*, and *clpP* genes. In general, most of the isolates harbored *nanA* (96.2%) and *piaA* (99.0%). Significant association was suggested between carriage rate and serotype in *cps2A, cbpA, pspA and nanA*. Besides, *cps2A, cbpA*, and *pspA* was also associated with clonal complex.

**Table 3:** Virulence genes among major serotypes and clonal complexes (%).

**Categories**	***cps2A***		***cbpA***		***pspA***		***nanA***		***piaA***		***lytA***		***ply***		***psaA***		***pavA***		***spxB***		***htrA***		***clpP***		**Total**	
	***n***	**%**	***n***	**%**	***n***	**%**	***n***	**%**	***n***	**%**	***n***	**%**	***n***	**%**	***n***	**%**	***n***	**%**	***n***	**%**	***n***	**%**	***n***	**%**	***n***	**%**
**Source**																										
CAP	111	45.7	107	44	83	34.2	234	96.3	241	99.2	243	100	243	100	243	100	243	100	243	100	243	100	243	100	243	84.7
HAP	16	36.4	20	45.5	14	31.8	42	95.5	43	97.7	44	100	44	100	44	100	44	100	44	100	44	100	44	100	44	15.3
		*P* = 0.252		*P* = 0.861		*P* = 0.763		*P* = 1.000		*P* = 0.394		-		-		-		-		-		-		-		
**Serotypes**																										
19F	6	6.3	83	86.5	7	7.3	96	100	95	99	96	100	96	100	96	100	96	100	96	100	96	100	96	100	96	33.4
6A	10	29.4	2	5.9	30	88.2	34	100	34	100	34	100	34	100	34	100	34	100	34	100	34	100	34	100	34	11.8
6B	4	17.4	0	0	18	78.3	23	100	23	100	23	100	23	100	23	100	23	100	23	100	23	100	23	100	23	8.0
19A	28	100	25	89.3	2	7.1	27	96.4	28	100	28	100	28	100	28	100	28	100	28	100	28	100	28	100	28	9.8
23F	13	54.2	3	12.5	7	29.2	20	83.3	24	100	24	100	24	100	24	100	24	100	24	100	24	100	24	100	24	8.4
14	24	100	24	100	4	16.7	24	100	23	95.8	24	100	24	100	24	100	24	100	24	100	24	100	24	100	24	8.4
		*p* = 0.000		*p* = 0.000		*p* = 0.000		*p* = 0.001		*p* = 0.597		-		-		-		-		-		-		-		
**Clonal complexes**																										
CC271	35	28.5	96	78.0	10	8.1	121	98.4	121	98.4	123	100	123	100	123	100	123	100	123	100	123	100	123	100	123	42.9
		*P* = 0.000		*P* 0.000		*P* 0.000		*P* = 0.169		*P* = 0.802		-		-		-		-		-		-		-		
**Total**	127	44.3	127	44.3	97	33.8	276	96.2	284	99.0	287	100	287	100	287	100	287	100	287	100	287	100	287	100	287	100

*cps2A* was present in 44.3% isolates and all serotypes 19A and 14 possessed it. The majority of serogroup 19 isolates carried *cbpA*, including 19F (86.5%) and 19A (89.3%). Serotypes 6A and 6B were the most dominant serotypes to carry *pspA*. The relationship between virulence patterns and serotypes of *S. pneumoniae* isolated from CAP and HAP was listed in Table 4. Based on the studied genes, the most common virulence pattern in current study was *lytA-ply-psaA-pavA-spxB-htrA-clpP-cbpA-nanA-piaA* (27.9%), with 19F accounting for the majority and 90% were MDR, followed by pattern *lytA-ply-psaA-pavA-spxB-htrA-clpP-cps2A-nanA-piaA* (16.7%) that contains a variety of serotypes.

**Table 4:** The relationship between virulence pattern and serotypes of *S. pneumoniae* isolated from CAP and HAP.

**Virulence pattern**	**Isolates (No.)**	**Proportion (%)**	**CAP (No.)**	**HAP (No.)**	**Related serotypes (No.)**
*lytA-ply-psaA-pavA-spxB-htrA-clpP-pspA*	1	0.3	0	1	untyped (1)
*lytA-ply-psaA-pavA-spxB-htrA-clpP-piaA*	1	0.3	1	0	Untyped (1)
*lytA-ply-psaA-pavA-spxB-htrA-clpP-cps2A-nanA*	1	0.3	1	0	14 (1)
*lytA-ply-psaA-pavA-spxB-htrA-clpP-cps2A-piaA*	1	0.3	1	0	33F (1)
*lytA-ply-psaA-pavA-spxB-htrA-clpP-pspA-piaA*	2	0.7	2	0	23F (2)
*lytA-ply-psaA-pavA-spxB-htrA-clpP-nanA-piaA*	29	10.1	22	7	19F (8),6A (3),6B (4),23F (5),15A (1),18 (1),untyped (7)
*lytA-ply-psaA-pavA-spxB-htrA-clpP-cbpA-nanA*	1	0.3	1	0	19F (1)
*lytA-ply-psaA-pavA-spxB-htrA-clpP-cps2A-pspA-piaA*	3	1.0	3	0	19A (1),untyped (2)
*lytA-ply-psaA-pavA-spxB-htrA-clpP-cps2A-nanA-piaA*	48	16.7	44	4	14 (19),23F (11),11A (1),15A (1),15B/C (4),19A (2),19F (3),20 (1),6B (1),9V (1),untyped (4)
*lytA-ply-psaA-pavA-spxB-htrA-clpP-cbpA-nanA-piaA*	80	27.9	67	13	19F (76),15A (2),6A (1),untyped (1)
*lytA-ply-psaA-pavA-spxB-htrA-clpP-cbpA-pspA-piaA*	1	0.3	1	0	23F (1)
*lytA-ply-psaA-pavA-spxB-htrA-clpP-pspA-nanA-piaA*	38	13.2	31	7	6A (19),6B (15),23F (3),19F (1),
*lytA-ply-psaA-pavA-spxB-htrA-clpP-cps2A-cbpA-pspA- piaA*	2	0.7	1	1	23F (1),untyped (1)
*lytA-ply-psaA-pavA-spxB-htrA-clpP-cps2A-cbpA-nanA-piaA*	29	10.1	23	6	19A (24),19F (1),23F (1),15B/C (1),11A (1),untyped (1)
*lytA-ply-psaA-pavA-spxB-htrA-clpP-cps2A-pspA-nanA-piaA*	36	12.5	31	5	6A (10),34 (6),14 (4),6B (3),7C (3),19F (1),15B/C (1), 11A (1),4 (1),untyped (6)
*lytA-ply-psaA-pavA-spxB-htrA-clpP-cbpA-pspA-nanA-piaA*	7	2.4	7	0	19F (4), 15A (2), 6A (1)
*lytA-ply-psaA-pavA-spxB-htrA-clpP-cps2A-cbpA-pspA-nanA-piaA*	7	2.4	7	0	19F (1),19A (1),34 (2),15B/C (1),untyped (2)

## Discussion

WHO reported that pneumonia accounts for 16% of all deaths of children under 5 years old, killing 920,136 children in 2015, with the most common cause of bacterial pneumonia being *S. pneumoniae*^2^. In this study, we identified a total of 287 pediatric patients diagnosed with pneumococcal pneumonia from January to December 2018 in Shanghai, China. Most of them (261,90.9%) were under 5 years old and diagnosed less in the summer, which was in line with the recent results from China (Cai et al., 2018). A male sexual superiority was also noticed among the study population, so as in other studies (Cai et al., 2018; Arushothy et al., 2019). In current study, chronic diseases were observed in 15.7% patients, which is an independent risk factor for pneumonia-related mortality in children (Zhang et al., 2013; Sonego et al., 2015; Nguyen et al., 2017). HAP has a higher rate of congenital heart disease than CAP, and more CAP with asthma. Meanwhile, we also noted almost half of children were coinfected with *Mycoplasma pneumoniae* or *Respiratory syncytial virus*, etc. Possibly because viral respiratory tract infections are a major facilitator of pneumococcal infections (Smith et al., 2014; Cawcutt and Kalil, 2017).

Our present study demonstrated that the most common serotypes among children in Shanghai were 19F, 6A,19A,23F,14,6B, and 34 and the serotype coverage of PCV 13 was 80.5%, which was similar to other recent studies in China but the ranking orders varied (Li et al., 2018; Shi et al., 2019). However, these serotypes were different from those in Latin America and the Caribbean (Gentile et al., 2012). PCV13 covered more isolates in Shanghai than in other countries (Miyazaki et al., 2017; Dalcin et al., 2018), probably because it was just licensed for optional use in 2016 and had not been taken into the standard childhood immunization program in mainland China. Compared with our previous reports (Pan et al., 2015a,b), the proportion of serotype 19A and 23F decreased while 14 and 34 increased. There was a reduction of 2–5% in serotype 15B/C, replaced by the appearance of 15A, mainly from CAP. As the methods for serotype in this study have technical limitations, 26 strains were identified as non-typeable, and most of them were from HAP. The PCV 13 coverage was a little lower than that prior to it was licensed in China, which may be on account of the selection of vaccines or natural fluctuations. Although the vaccine is not widely used, we still observed the phenomenon of serotype changes, which suggests that the changes in serotype maybe not directly related to vaccination. Studies indicated that pneumococci was able to change their capsular serotype by exchanging the capsular locus genes (Coffey et al., 1998). In our study, the serotype coverage of PCV13 remains much higher than the average rate of other regions in China (68.4%) (Chen et al., 2018). Hence, vaccination is of great importance to eliminate the burden of pneumococcal infection in Shanghai. Nevertheless, other studies discussed serotypes rates within the vaccine will decrease by 50% due to PCV13, which becomes a problem (Shiri et al., 2017; Suzuki et al., 2017). There were rapid and substantial reductions of disease caused by PCV-serotypes (children aged <5 years old) in Australia, Canada, England and Wales, South Africa and the USA after the introduction of pneumococcal vaccines, subsequently an increase in the incidence of diseases caused by non-PCV7 serotypes (Pneumococcal vaccines WHO position paper, 2012). And that the distribution of serotypes vary across different affected populations as well as economic development, and change over time (Johnson et al., 2010; Zhao et al., 2017; Yan et al., 2019), so these vaccines should be reevaluated systematically and monitored long-term.

Penicillin represents as the first choice for the antibiotic treatment of *S. pneumoniae* infections, but the resistance to it has continued to increase across the world (Linares et al., 2010). Its susceptibility rates varied from 70.7% in Europe to 52.4% in Asia-Pacific region for all years combined from 1997 to 2016 (Sader et al., 2019). In comparison with previous study (Pan et al., 2015b), there is a significant increase in the proportion of PNSP (from 20 to 31.7%), which means approximately one third of the isolates in this study were non-susceptible to penicillin. In addition, decrease in erythromycin susceptibility was observed as well, and compared with North America whose susceptibility rate was 55.0–56.0%, its resistance is very severe in our study (Sader et al., 2019). It was reported that the susceptibility rates were lower among isolates from pediatric patients than adults for penicillin and azithromycin (Sader et al., 2019). In the Chinese consensus, antibiotics use in children is restricted due to the concern for safety and risk of adverse events. For instance, despite the widely use of fluoroquinolones for their highly effective in adults, their use in children is limited because of the side-effect and toxicity they may cause, such as destructive arthropathy and influence on the central nervous system (Patel and Goldman, 2016). The increase resistance maybe attributed to the selection pressure caused by the widespread use of β-lactam and macrolide antibiotics that were used as first-line therapy in children for their little side-effect and lower toxicity (Bradley et al., 2011). HAP with a higher rate of PNSP and proportion of MDR than CAP was noticed in this study, which suggested that the strains within the community were more susceptible and the strains obtained within hospital were more resistant. The higher resistance in HAP may be due to the higher rates of PCV13/non-PCV7 serotypes because of the significant relationship between antibiotic resistance and serotypes. Our data showed that PCV13 covered 85.1% MDR isolates, which suggested vaccine has the potential to control the spread of MDR (Maraki et al., 2010, 2018). Against all isolates vancomycin, linezolid and moxifloxacin exhibited good activity, which could be alternatives for treatment of PRSP and MDRSP infections.

The most common STs in this study was ST271,ST320,ST3173, and ST876, which was similar to other reports in other regions of China (Li et al., 2018; Shi et al., 2019; Yan et al., 2019). When comparing with the multi-center study in Shanghai in 2013 (Pan et al., 2015b), we observed that the dominating STs were basically the same, with a reduction in ST81 and increase in ST3173 and ST876. The rate of clones registered as MDR PMEN clones was essentially equal but different in content composition. Except for four common international antibiotic-resistant clones Taiwan^19F^-14, Spain^23F^-1, Spain^6B^-2 and Taiwan^23F^-15 in Shanghai, Sweden^15A^-25 was newly observed in our study. After the introduction of PCV13, the increasing serotype 15A was noted in Norway, Canada and Japan (Steens et al., 2013; Ricketson et al., 2014; Naito et al., 2016). In our study, six isolates belongs to Sweden^15A^-25/ST63 and its single locus variant. Most of the clones in 2018 carried the same serotypes in 2013. In addition, ST2248, a SLV of Sweden^15A^-25/ST63, were detected in serotype 14. Five new STs were also obtained, related to serotype 34, 6A/B and 15B/C. With the popularization of vaccines, a careful monitoring should be required in the future about the Sweden^15A^-25/ST63 known as one of the MDR PMEN clones (Hackel et al., 2013).

Dispensable genes, not required for bacterial growth, provide survival advantages to *S. pneumoniae*. We detected the prevalence of a total of 12 virulence factors of particular important. These genes play a role in adherence to host cells, evasion of host immune responses and promotion of the biofilms formation to enhance bacterial survival competitiveness (Brooks and Mias, 2018). Comparison with other investigations, the detection rate varied across the regions and isolates disease related or colonization-related (Qin et al., 2013; Fu et al., 2019). Our data indicated that there is no difference between CAP and HAP isolates in the presence and distribution of virulence genes. The positive rate of *cps2A, cbpA, pspA*, and *nanA* varied from different serotypes. It's worth noting that nine isolates were negative for the housekeeping gene *cpsA* similar to other reports. Studies indicated that non-encapsulated *S. pneumoniae* could also cause 3–19% of pneumococcal diseases and the current conjugate vaccines may be ineffective against them because of serotype specificity (Keller et al., 2016). The appearance of serotype replacement and non-encapsulated strains suggested a new type vaccine should be designed to prevent pneumococcal infections. Virulence genes are crucial candidate targets for the development of next-generation protein vaccines. For instance, a study reported that generate neutralizing antibodies immunization with pneumococcal neuraminidases *nanA, nanB*, and *nanC* was able to increase survival in mice (Janapatla et al., 2018). Our results showed that there is a high prevalence in *nanA, piaA, lytA, ply, psaA, pavA, spxB, htrA and clpP* whereas lower in *cps2A, cbpA*, and *pspA*. A requisite for a vaccine candidate is that the selected gene (s) is widely distributed in the target pneumococcal population (Cornick et al., 2017). The extremely high carriage rate suggests the potential to develop vaccines. However, this current study suggests that *cps2A, cbpA, and pspA* might be not suitable to be candidates for vaccines in Shanghai.

In conclusion, our study described the epidemiology characteristics of *S. pneumoniae*. from children with pneumonia in Shanghai. 19A/F, 6A/B, 23F and 14 were identified as the predominating serotypes in 2018. PCV13 serotype coverage was a little reduced than before. Compared with CAP, isolates from HAP had more PCV13/non-PCV7 serotypes, higher rate of PNSP and higher proportion of MDR. Moreover, our results revealed the type of clonal disseminations, and more attention should be paid to the emerging Sweden^15A^-25/ST63 with the popularization of vaccines. *lytA, ply, psaA, pavA, spxB, htrA* and *clpP* were observed in all isolates, which may be candidates for next generation vaccines. Finally, long-term high-quality surveillance should be conducted to assess impact and effectiveness brought by vaccines, and provide a foundation for prevention strategies and vaccine policies.

## Data Availability

All datasets generated for this study are included in the manuscript.

## Author Contributions

HZ, WZ, and FP conceived and designed the experiments. WZ and YYS performed antibiotic susceptibility testing and serotyping. WZ and BW performed MLST and virulence genes detection. HZ, YS, TZ, and CW contributed strains and case data collection. WZ wrote the first draft of the manuscript, and all authors contributed to manuscript revision, read and approved the submitted version.

### Conflict of Interest Statement

The authors declare that the research was conducted in the absence of any commercial or financial relationships that could be construed as a potential conflict of interest.

## Footnotes

^1^Prevention CfDCa. *Causes of Pneumonia*. Available online at: https://www.cdc.gov/pneumonia/causes.html.

^2^World Health Organization. *Pneumonia-Key facts*. Available online from: https://www.who.int/news-room/fact-sheets/detail/pneumonia.
